# Chronic low back pain and its impact on physical function, mental health, and health-related quality of life: a cross-sectional study in Singapore

**DOI:** 10.1038/s41598-022-24703-7

**Published:** 2022-11-21

**Authors:** Lixia Ge, Michelle Jessica Pereira, Chun Wei Yap, Bee Hoon Heng

**Affiliations:** Health Services & Outcomes Research, National Healthcare Group, 3 Fusionopolis Link, #03-08 Nexus@One-North (South Lobby), Singapore, 13854 Singapore

**Keywords:** Health care, Risk factors

## Abstract

Chronic low back pain, defined as low back pain lasting more than 3 months, is a globally prevalent health problem with significantly high medical and economic burden on individuals and the society. This study aimed to estimate the prevalence of chronic low back pain and examine its association with health outcomes including physical function, mental health, and quality of life among adult population in Singapore. Cross-sectional secondary data analysis was performed using baseline data of the 1941 adults (mean age: 52.6 years, range: 21–97 years) from a representative population health survey conducted in the Central region of Singapore. Those with self-reported chronic low back pain in past six months were identified. The Late-Life Function and Disability Instrument, Patient Health Questionnaire-9, and EQ-5D-5L were used to measure physical function and limitation, mental health, and health-related quality of life, respectively. Generalized Linear Regressions were used to examine the association of chronic low back pain with physical function, limitation, depressive symptoms, and health-related quality of life. There were 8.1% (n = 180) participants reporting having chronic low back pain in past six months, among whom 80.5% sought treatments at either primary care, specialist outpatient, or Traditional Chinese Medicine clinics. Individuals with chronic low back pain reported poorer physical function, more limitations in performing major life tasks and social activities, more depressive symptoms, and lower health-related quality of life (all p < 0.01), even after adjusting for socio-demographics, lifestyle factors, and number of morbidities. The prevalence of chronic low back pain was 8.1% among the study population. Chronic low back pain was associated with poorer physical function, more limitations and depressive symptoms, and lower health-related quality of life. The findings highlight the significant impact of chronic low back pain on physical function and limitation, mental health, and health-related quality of life in a general population. Increased awareness on prevention, early and proper management of low back pain, and rehabilitation policies are required to better tackle the burden of low back pain at the population level.

## Introduction

Low back pain (LBP) is a common musculoskeletal problem with high prevalence among middle-aged and older adults^1,2^. It was estimated that 49–90% of people in developed countries will develop LBP at some point in their life^3^. As the main contributor to the overall burden of musculoskeletal conditions, LBP remains the leading global cause of years of life with disability (YLDs) in 2019 worldwide^4,5^. In Singapore, musculoskeletal disorder is the leading cause of YLDs and LBP accounted for 9.5% of total YLDs: 10.3% among those aged 15–49 years, 10.1% among those aged 50–69 years old and 7.7% among those aged 70 years and above in 2019^6^. LBP has become a great public health issue resulting in considerable medical and economic burden (including direct and indirect costs) to individuals, families, and the society^7^.

There have been many studies on the epidemiology of LBP in the past several decades. However, the accurate assessment of the prevalence remains challenging as there is little scientific evidence on LBP diagnosis^3^. Due to the heterogeneity of study population, design and methodology, the prevalence derived from different studies may not be directly comparable^8^. However, studies worldwide have consistently documented that LBP has significant impacts on individuals’ physical and psychosocial health. It not only limits one’s physical functioning and daily activities^9,10^, but also has the potential to cause higher levels of subclinical distress and depression^11,12^. A local study on LBP patients attending the Musculoskeletal Physiotherapy Clinic in a government-structured hospital also found that LBP results in significant disability and time lost off work^13^. When LBP persists for 12 weeks or longer, it becomes chronic low back pain (cLBP)^14^. CLBP may have more profound impacts on one’s performance of social responsibilities in family, work and social life, which may significantly affect the health-related quality of life (HRQoL)^15,16^.

In view of the significant burden of LBP (as estimated by YLDs), it is important to estimate the prevalence of cLBP, examine the potential risk factors, and evaluate its association with health outcomes among community-dwelling adults in Singapore. To date, there is scarcity of research on cLBP among general population in Singapore. To address this gap, we conducted this cross-sectional secondary data analysis by using population-based health survey data collected in the Central region of Singapore to (1) estimate the prevalence of cLBP in the community-dwelling adult population and healthcare seeking rates; (2) examine the association between cLBP and socio-demographic, lifestyle, and health factors, and (3) examine the association between cLBP and physical function and limitation, depressive symptoms, and HRQoL. We hypothesized that cLBP would have a negative impact on physical function and limitation, depressive symptoms, and HRQoL.

## Methods

### Study design and participants

Cross-sectional secondary data analysis was performed using baseline data of the longitudinal Population Health Index (PHI) study, a population health survey conducted in the Central region of Singapore among representative community-dwelling adults. The participant recruitment for the PHI study was initiated in November 2015 and ended in January 2017, followed by two subsequent annual follow-ups. Singapore citizens and permanent residents who were aged 21 years and above and staying in any of the nine planning areas (PAs, including Novena, Geylang, Kallang, Rocher, Toa Payoh, Bishan, Serangoon, Ang Mo Kio, and Hougang) of the Central region of Singapore were eligible for the study. The estimated population in the Central region was 821,650 residents^17^.

The study design, sampling procedure, participant recruitment, and follow-up processes of the PHI survey were detailed elsewhere^17,18^ and the contents of the survey questionnaire were listed in another study^19^. In brief, a total of 5350 residential dwelling units from the nine PAs were proportionally selected from the National Database on Dwellings in Singapore maintained by the Department of Statistics. An invitation letter was sent to the selected dwelling units to notify and invite them to participate in the survey. During the house visits to the selected dwelling units by trained interviewers, Kish tables were used to identify one eligible household member from each dwelling unit to participate in the study. A total of 1942 individuals (response rate: 53.3%, based on 3645 eligible residents) were recruited and participated in the baseline survey.

All participants who responded to the question on cLBP at baseline (N = 1941) was sampled for this secondary data analysis. To ensure the sufficiency of this sample size for the study, we calculated the minimum number of necessary sample for prevalence studies using Scalex SP Version 1.0.1^20^. The calculation result suggested that for the expected prevalence of 13.16%^4^, the required sample size was 1098 for the margin of error of ± 2% in estimating the prevalence with 95% confidence and considering the potential non-response rate of 50%. Hence, the actual sample size used in this study was sufficient.

### Measures

#### Chronic low back pain status

Presence of cLBP was determined based on responses to the following question included in the PHI survey: “In the past 6 months, have you ever sought medical consultation/treatment for chronic low back pain (lasting for more than three months)?”. There were five options. An individual without cLBP would choose the option “No pain, not applicable” and those with cLBP but never sought medical consultation/treatment would select “With pain but never sought treatment”. Individuals with cLBP and sought medical consultation/treatment in past six months were asked to indicate where they received the consultation/treatment from: “Polyclinic/General Practitioner (GP)”, “Specialist Outpatient clinic (SOC)”, or “Traditional Chinese Medicine (TCM) Clinic”. Two or more options could be selected for the care setting. Based on their response to the question, the individuals were then categorized into either group: “without cLBP” or “with cLBP”.

#### Physical function and limitation

Overall physical function refers to the “ability to perform discrete actions or activities as part of daily routines without the help of others”^21^. The overall physical function was measured using the 32-item Function Domain of the Late-Life Function and Disability Instrument (Late-Life FDI) which include items measuring upper extremity functioning (7 items), basic lower extremity functioning (14 items), and advanced lower extremity functioning (11 items)^21^. The questions were phased “How much difficulty do you have (doing a particular activity) without the help of someone else and without the use of assistive devices?” with five response options of “5 = None”, “1 = A little”, “3 = Some”, “4 = Quite a lot”, and “5 = Cannot do”.

Individuals’ physical limitation in performing major life tasks and social activities in the community was assessed by the 16-item Limitation domain of the Late-Life FDI. The limitation questions were phrased “To what extent do you feel limited in (doing a particular task)?” and each question had five response options: “5 = Not at all”, “4 = A little”, “3 = Somewhat”, “2 = A lot”, and “1 = Completely”.

The raw scores of the Function and Limitation domains were calculated by summing up the respective item scores. They were then transformed to scaled scores ranging from 0 to 100 with 0 indicating worse function or more limitation and 100 indicating better overall function or less limitation^21^. The Late-Life FDI demonstrated high level of reliability with Cronbach’s alphas for Overall Function and Limitation of 0.97 and 0.94, respectively in the present study.

#### Mental health

The 9-item Patient Health Questionnaire (PHQ-9), a self-administered version of the Primary Care Evaluation of Mental Disorders diagnostic instrument for common mental disorders, was used to assess mental health^22^. A 4-point scale (0 = not at all, 1 = several days, 2 = more than half the days, 3 = nearly every day) was used to assess each PHQ-9 item. The score of each item was summed up to derive the total depressive symptoms score which ranges from 0 to 27, with a higher score indicating more or severer depressive symptoms^22^. As a well validated and widely used brief diagnostic and severity measure of depression, the PHQ-9 also demonstrated good internal consistency reliability (Cronbach's alpha = 0.77) in this study.

#### Health-related quality of life

HRQoL was measured using the EQ-5D-5L descriptive system of five domains (mobility, self-care, usual activities, pain/discomfort, and anxiety/depression) with five levels (level 1 = no problems, level 2 = slight problems, level 3 = moderate problems, level 4 = severe problems and level 5 = extreme problems)^23^. A single index value (EQ-5D index score) was derived from the EQ-5D-5L descriptive system, with 0 indicating death and 1 (the highest score) indicating complete health. An EQ-5D index score less than 0 indicated health state worse than death. As there was no available EQ-5D-5L value set for Singapore, the Japan value set^24^ was used to calculate the EQ-5D index score to represent a person’s HRQoL. The EQ visual analogue scale (VAS) records a person’s self-rated health on a vertical scale ranging from 0 (the worst health you can imagine) to 100 (the best health you can imagine). The EQ-5D-5L demonstrated acceptable internal consistency reliability in the present study with a Cronbach’s alpha of 0.77.

#### Other data collected

The socio-demographic factors used in the analysis included age in years, sex, marital status (single, married, and divorced/widowed), living arrangement (living alone vs living with others), and financial status assessed by self-reported money insufficiency for basic daily living (perceived money sufficiency vs insufficiency). Occupation was collected using the ten major groups described in the Singapore Standard Occupational Classification 2015^25^ and re-categorized into three groups: PMETs (Professionals, Managers, Executives and Technicians, including Major Group 1–3, X), CSSWs (Clerical, Sales and Service Workers, including Major Group 4–5), and PTOCLs (Production and Transport Operators, Cleaners and Labourers, including Major Group 6–9). An additional group named “Others” was added to the occupational groups to represent individuals who were unemployed and inactive. In addition, lifestyle factors including smoking status (never smoked, former smoker, and current smoker) and alcohol misuse (yes and no, assessed by the Alcohol Use Disorders Identification Test Consumption screening tool^26^) were also included. Furthermore, the number of diagnosed chronic morbidities (0, 1, and 2 or more) was derived based on self-reported diagnosis of the 17 chronic diseases including dyslipidemia, high blood pressure, diabetes, chronic kidney disease, heart attack/ischemic heart disease, heart failure, stroke/transient ischemic attack, asthma, chronic bronchitis/emphysema/chronic obstructive pulmonary disease, cancer, osteoarthritis/gout/rheumatoid arthritis, osteoporosis, depression, anxiety disorder, schizophrenia, dementia/Alzheimer’s, and Parkinson’s disease^27^.

### Data analysis

Characteristics of the study participants were described using mean and standard deviation (SD) for continuous variables, and frequency and weighted percentage for categorical variables. The weighted prevalence of cLBP for all participants and by individual socio-demographics, lifestyle and health categories were calculated. The weighted rates of seeking care at different care settings for all participants were reported. The differences in socio-demographics, lifestyle, and health characteristics between participants with and without cLBP were examined using chi-square tests for categorical variables and t-test for continuous variables. A logistic regression anaysis was performed to determine the factors associated with cLBP (dependent variable). Odds ratios (OR) and 95% confidence intervals (95% CI) were reported.

Health outcomes including physical function and limitation, depressive symptoms, and HRQoL were described using mean ± SD, median, and the first (Q1) and third quartiles (Q3). They were compared between participants with and without cLBP using chi-square tests for categorical outcome variables and Mann Whitney U tests for continuous outcome variables. As health outcome variables were generally skewed towards larger values, a series of Generalized Linear Models (GLM) with Gamma family distribution and log link function were performed to determine the association between cLBP (independent variable) and each health outcome (dependent variable), adjusting for socio-demographics (including age in years, sex, marital status, living arrangement, occupational groups, and financial status), lifestyle factors (including smoking status and alcohol misuse), and number of diagnosed chronic morbidities. Beta coefficients (B) and 95% CIs were reported. Any participant with missing data were omitted from the regression models. The number of records with missing data for the outcome variables were: Overall Function (n = 1, 0.05%), PHQ-9 depressive symptom score (n = 11, 0.57%), EQ5D anxiety and EQ5D Index (n = 4, 0.21%), and EQ VAS (n = 7, 0.36%). Variables in each model were tested for multicollinearity and those with a variance inflation factor (VIF) value of 5 or higher^28^ would be removed from the respective model.

All the analyses were conducted using Stata/SE 16.1 for Windows (StataCorp, College Station, TX) and a p value of 0.05 was set as the level of significance for all tests.

### Ethics approval and consent to participate

The PHI study was approved by the ethics review committee of the National Healthcare Group Domain Specific Review Board (Reference Number: 2015/00269). The study was conducted in accordance with the Declaration of Helsinki. Written informed consent was obtained from all individual participants after they were being informed about the study objectives and the safeguards put in place so that confidentiality of the collected data is maintained.

## Results

### Characteristics of participants

The characteristics of all the 1941 participants were described in Table 1. The mean age of the participants was 52.5 years old with a standard deviation of 16.9 years. Majority of the participants were Chinese (78.3%), 56.2% were females, and 36.9% were unemployed or inactive.

**Table 1:** Characteristics of all participants and by chronic low back pain status.

Characteristics	Total (N = 1941)		cLBP prevalence	cLBP status				
				Without cLBP (n = 1761)		With cLBP (n = 180)		p-value
	n/mean	Weighted % /SD		n/mean	Weighted %/SD	n/mean	Weighted %/SD	
**Age (Mean, SD)**	*52.6*	16.9		52.1	16.7	57.2	17.5	** < 0.001**
**Age groups (n, %)**								** < 0.001**
21–39	499	29.6	5.2	469	30.5	30	18.8	
40–59	741	36.3	7.7	676	36.5	65	34.6	
60–74	490	24.2	8.9	442	24.0	48	26.6	
75 & above	211	9.9	16.4	174	9.0	37	20.0	
**Gender (n, %)**								0.093
Male	859	43.8	7.1	790	44.3	69	38.5	
Female	1082	56.2	8.9	971	55.7	111	61.5	
**Ethnicity (n, %)**								0.417
Chinese	1523	78.3	8.3	1380	78.1	143	80.1	
Malay	154	8.2	8.8	136	8.2	18	8.9	
Indian	211	11.1	6.3	197	11.3	14	8.6	
Others	53	2.4	8.3	48	2.4	5	2.5	
**Highest education level (n, %)**								**0.001**
No formal education	286	14.2	14.0	241	13.3	45	24.4	
Primary	226	11.4	7.7	206	11.5	20	10.8	
Secondary	597	28.9	7.2	545	29.2	52	25.5	
Post-secondary and above	832	45.5	7.0	769	46.1	63	39.3	
**Marital status (n, %)**								** < 0.001**
Single	455	25.3	5.4	422	26.1	33	16.7	
Married/cohabiting	1175	63.5	7.8	1079	63.8	96	60.9	
Divorced/widowed	311	11.2	16.2	260	10.2	51	22.4	
**Occupational groups (n, %)**								**0.005**
PMETs	586	31.2	5.8	550	32.0	36	22.5	
CSSWs	397	20.1	7.7	363	20.2	34	19.1	
PTOCLs	243	11.8	10.0	217	11.5	26	14.6	
Others (unemployed and inactive)	715	36.9	9.6	631	36.3	84	43.9	
**Living arrangement (n, %)**								**0.036**
Living alone	221	5.3	12.4	192	5.1	29	8.2	
Living with others	1720	94.7	7.9	1569	94.9	151	91.8	
**Self-reported money sufficiency (n, %)**								** < 0.001**
Sufficient	1651	85.7	6.5	1526	87.2	125	68.6	
Insufficient	290	14.3	17.8	235	12.8	55	31.4	
**Smoking status (n, %)**								0.447
Never smoked	1445	76.2	7.5	1318	76.7	127	70.4	
Former smoker	238	11.4	10.2	212	11.1	26	14.3	
Current smoker	258	12.4	10.0	231	12.2	27	15.4	
**Alcohol misuse (n, %)**								0.153
No	1,434	73.4	8.6	1,293	73.0	141	77.4	
Yes	507	26.6	6.9	468	27.0	39	22.6	
**Number of chronic morbidities (n, %)**								** < 0.001**
0	835	44.6	3.8	799	46.7	36	21.0	
1	390	20.4	9.7	349	20.0	41	24.3	
2 or more	716	35.0	12.7	613	33.3	103	54.7	

Significant values are in bold.

*cLBP* chronic low back pain, *PMETs* Professionals, Managers, Executives and Technicians, including Major Group 1–3, X), *CSSWs* Clerical, Sales and Service Workers, including Major Group 4–5, *PTOCLs* Production and Transport Operators, Cleaners and Labourers, including Major Group 6–9.

The weighted % were column percentages.

### Prevalence of chronic low back pain and rates of care seeking

There were 180 participants (weighted percentage: 8.1%) reported having cLBP in past 6 months. Among the 180 participants reported having cLBP, 143 (80.5%) sought consultations or treatments at polyclinics/GPs (46.2%), SOCs (43.5%) or TCM clinics (40.1%) in past 6 months, and 20.7% sought consultations or treatments at two or more of these three care settings.

### Socio-demographics associated with chronic low back pain

Compared to those who did not report cLBP (n = 1761), individuals reporting cLBP (n = 180) were significantly older (mean ± SD: 57.2 ± 17.5 vs 52.1 ± 16.7) with higher proportion of individuals aged 75 years and above (20.0% vs 9.0%), and more likely to be divorced/widowed (22.4% vs 10.2%) or living alone (8.2% vs 5.1%), more likely to report having no formal education (24.4% vs 13.3%), money insufficiency (31.4% vs 12.8%) or having two or more chronic conditions (54.7% vs 33.3%) (Table 1). After adjusting for the rest of the categorical socio-demographic factors showed in Table 1, individuals who were females (OR 1.55, 95% CI 1.04, 2.32), perceiving money insufficiency for basic daily living (OR 2.71, 95% CI 1.84, 3.99), and having existing comorbidity (OR 3.36, 95% CI 2.09, 5.41) had higher risk of cLBP (Table 2).

**Table 2:** Association between socio-demographics and chronic low back pain using logistic regression.

Characteristics	Odds ratio	95% confidence interval	p-value
**Age groups** (ref. 21–39)			
40–59	1.09	0.64, 1.84	0.761
60–74	0.96	0.49, 1.86	0.901
75&above	1.50	0.67, 3.38	0.323
**Female (ref. male)**	1.55	1.04, 2.32	**0.031**
**Ethnicity (ref. Chinese)**			
Malay	1.11	0.63, 1.97	0.717
Indian	0.59	0.33, 1.07	0.085
Others	0.73	0.27, 1.97	0.529
**Highest education level (ref. no formal education)**			
Primary	0.72	0.39, 1.32	0.286
Secondary	0.96	0.58, 1.60	0.886
Post-secondary & above	1.62	0.88, 2.97	0.123
**Marital status (ref. single)**			
Married	1.13	0.70, 1.82	0.622
Divorce/widowed	1.72	1.00, 2.98	0.051
**Occupational groups (ref. PMETs)**			
CSSWs	1.22	0.71, 2.10	0.466
PTOCLs	1.56	0.82, 2.97	0.178
Others (Unemployed and inactive)	1.16	0.68, 2.00	0.585
**Live alone (ref. living with others)**	1.25	0.76, 2.05	0.387
**Self-reported money insufficiency (ref. sufficient)**	2.71	1.84, 3.99	** < 0.001**
**Smoking status (ref. never smoked)**			
Former smoker	1.31	0.78, 2.19	0.300
Current smoker	1.32	0.77, 2.26	0.320
**Alcohol misuse (ref. no)**	0.97	0.64, 1.48	0.898
**Number of chronic morbidities (ref. 0)**			
1	2.73	1.68, 4.43	** < 0.001**
2 or more	3.36	2.09, 5.41	** < 0.001**

Significant values are in bold.

*PMETs* Professionals, Managers, Executives and Technicians, including Major Group 1–3, X), *CSSWs* Clerical, Sales and Service Workers, including Major Group 4–5; *PTOCLs* Production and Transport Operators, Cleaners and Labourers, including Major Group 6–9.

### Association between chronic low back pain and health outcomes

The comparison of physical function and limitation, mental health (depressive symptoms), and HRQoL between individuals with and without cLBP in past six months showed that those with cLBP had significantly lower overall function, more limitation, and more depressive symptoms. Individuals experienced cLBP had significantly higher proportions of individuals having problem in morbidity, self care, activity, or having pain or anxiety compared to those who did not have cLBP (Table 3). Individuals with cLBP also had lower HRQoL (lower EQ-5D index score and EQ5D VAS). Although those sought consultation/treatment for cLBP had relatively lower mean scores in overall function, limitation, and HRQoL, and higher depressive symptom score compared to those did not seek consultation/treatment, these differences were not significant (all p > 0.05).

**Table 3:** Comparison of health outcomes between individuals with and without chronic low back pain.

Health outcomes	Total		cLBP status				p-value
			Without cLBP		With cLBP		
	Mean ± SD / n(%)	Median (Q1–Q3)	Mean ± SD / n(%)	Median (Q1–Q3)	Mean ± SD / n(%)	Median (Q1–Q3)	
**Physical function and limitation**							
Overall function	84.0 ± 17.8	90.3 (73.4–100)	85.1 ± 17.0	90.3 (73.4–100)	73.6 ± 21.3	72.3 (58.6–100)	< 0.001*
Total Limitation	82.1 ± 17.3	83.4 (70.2–100)	82.7 ± 16.8	83.4 (70.2–100)	76.6 ± 20.5	74.8 (65.2–100)	< 0.001*
**Depressive symptoms**	1.1 ± 2.3	0 (0–1)	1.0 ± 2.1	0 (0–1)	2.6 ± 3.5	1 (0–4)	< 0.001*
**Health-related quality of life (EQ-5D-5L)**							
Problem in morbidity	138 (7.0)	–	102 (5.9)	–	36 (19.6)	–	< 0.001**
Problem in self care	49 (2.5)	–	37 (2.2)	–	12 (6.1)	–	< 0.001**
Problem in activity	113 (5.7)	–	82 (4.7)	–	31 (16.0)	–	< 0.001**
Problem in pain	415 (20.9)	–	315 (17.9)	–	100 (54.9)	–	< 0.001**
Problem in anxiety	93 (4.7)	–	69 (4.0)	–	24 (13.2)	–	< 0.001**
EQ-5D index	0.94 ± 0.13	1 (1–1)	0.94 ± 0.12		0.85 ± 0.16	0.81 (0.74–1)	< 0.001*
EQ-5D VAS	78.1 ± 14.5	80 (70–90)	79.0 ± 13.7		69.5 ± 18.2	70 (60–80)	< 0.001*

Significant values are in bold.

*cLBP* chronic low back pain, *Q1* the first quartile, *Q3* the third quartile, *VAS* Visual Analogue Scale.

*Mann–Whitney tests, **Chi-squared tests.

The GLM regression analysis results in Table 4 showed that cLBP remained significantly associated with lower overall physical function (B = − 0.08, 95% CI − 0.10, − 0.05), more physical limitation (B = − 0.04, 95% CI − 0.07, − 0.01), more or severer depressive symptoms (B = 0.63, 95% CI 0.32, 0.94), and worse HRQoL (EQ-5D Index: B = − 0.07, 95% CI − 0.09, − 0.05, EQ VAS: B = − 0.10, 95% CI − 0.13, − 0.07), even after adjusting for covariates (age was excluded from the models due to collinearity). The full generalized linear regression results including all adjusted variables are presented in Supplementary Table S1.

**Table 4:** Association between chronic low back pain and health outcomes using generalized linear models.

Health outcomes	With cLBP		
	Coeff.	95% CI	p-value
Overall function	− 0.08	− 0.10, − 0.05	** < 0.001**
Total limitation	− 0.04	− 0.07, − 0.01	**0.016**
Depressive symptoms	0.63	0.32, 0.94	** < 0.001**
EQ-5D index	− 0.07	− 0.09, − 0.05	** < 0.001**
EQ VAS	− 0.10	− 0.13, − 0.07	** < 0.001**

Significant values are in bold.

*cLBP* chronic low back pain, *Coeff.* beta coefficient, *95% CI* 95% confidence interval.

Variables adjusted in all the models were: sex, marital status, living arrangement, occupation groups, and financial status, smoking status, alcohol misuse, and number of diagnosed chronic morbidities. Age was excluded from the model due to collinearity.

## Discussion

Chronic low back pain is a major public health issue with considerably high medical, social, and economic impacts. This study estimated the prevalence of cLBP and rates of care in different settings, identified the potential factors associated with cLBP and examined the association of cLBP with health outcomes in representative community-dwelling adults in the Central region of Singapore. The results showed that the prevalence of cLBP was 8.1% in the study population, of whom 80.5% sought consultation/treatment at polyclinics/GPs, SOCs, or TCM clinics. The study identified significant association of cLBP with physical function and limitation, mental health, and HRQoL in the context of a community setting in Singapore.

The prevalence of cLBP in the study population was 8.1%, which is slightly higher than 7.5% – the estimated global age-standardized point prevalence of LBP (ranging from 3.92% in East Asia to 13.47% in Latin America) in 2017^4^. Existing evidence on the prevalence of LBP in different populations varies widely. The point-prevalence of LBP based on general population surveys in four high-income western countries (Britain, Belgium, Germany, and Sweden) ranged from 14% (Britain) to 35% (Sweden)^29^. One literature review reported that the one-year prevalence of LBP among adults was estimated to range from 15 to 20%^8^. A population-based study conducted in Taiwan reported that 25.7% reported LBP within the past three months^30^. It is also worth highlighting that these studies reported the prevalence of LBP without differentiating acute or chronic condition. In our study, we only identified cLBP (lasting for more than three months) in past six months. Hence, the prevalence of LBP in our study population (8.1%) was lower than the age-standardized point prevalence of LBP estimated for high-income Asia Pacific countries (13.16%)^4^, however, it was much higher than the prevalence reported in North Carolina population (3.9%) more than 20 years ago^31^. The heterogeneity in study population, definition or inclusion criteria for LBP, data source, measure used (point or lifetime prevalence, pooled, or age-standardized prevalence), and duration of observation across studies^32^ might explain the wide variation in LBP or cLBP prevalence across countries.

Among individuals who reported having cLBP in past 6 months, 80.5% sought consultation/treatment at different care settings with comparable rates ranging from 40.1% at TCM clinics to 46.2% at polyclinics/GPs. Unfortunately, care seeking in any emergency department of hospitals was not surveyed. The physician visit rate for LBP in past year was 61.2% in Spain^33^ while the pooled health-care utilisation rates for LBP from any health-care provider as reported in a systematic review was 67% in USA, 48% in Europe, and 47% in UK^32^. The higher care seeking rate observed in our study could be potentially explained by the higher distress caused by cLBP compared to acute LBP.

Consistent with the global trend that females had higher prevalence of cLBP than males^4^, the prevalence of cLBP in female adults in our population was 8.9%, which was slightly but not significantly higher than that in males (7.1%, p = 0.093). However, after adjusting for other socio-demographics, lifestyle factors and number of chronic morbidities, females had higher odds of cLBP. This study found cLBP was prevalent in the elderly aged 75 years and above and increasing age was associated with a higher odd of cLBP in univariate analysis, however, the association was no longer significant after adjusting for other covariates. Smoking^34^, lower education, and occupation^30^ were found to be risk factors for the development of LBP. Prior studies found that LBP is highly prevalent among agricultural/forestry/fishery workers^35^ and less well educated individuals^36,37^. Our study found individuals who were PTOCLs (blue-collar workers) and having no formal education had higher prevalence of cLBP, however, they were no longer associated with cLBP after controlling for the covariates. Consistent with other studies^38,39^, our study further confirmed that lower subjective economic situation (measured by self-reported money insufficiency for basic daily living in this study) and having multiple chronic morbidities were significantly associated with cLBP, even after adjusting for other covariates.

Prior studies have documented that LBP was associated with both physical function and mental wellbeing^11,40–42^. Although this study did not capture current existing or acute LBP which lasted for less than 3 months, we still observed significant association between cLBP and physical function, limitation, and depressive symptoms even after controlling for socio-demographics, lifestyle factors, and number of morbidities. The findings further showed that cLBP corresponded to reporting problems in all domains of HRQoL as measured by EQ-5D-5L. Consistent with the findings from a French study^43^, the association between cLBP and poorer HRQoL remained after controlling for socio-demographics, lifestyle and number of morbidities. Our findings underscore the significant impacts of cLBP on health outcomes and highlight the necessity of raising public awareness on LBP prevention and proper treatment. Although one local study found the association of occupational groups with physical and mental health^44^, it was not observed in our study.

To our knowledge, this is the first study estimating the prevalence of cLBP and care seeking rates and examining the association of cLBP with various health outcomes in community-dwelling adult population in Singapore. The sampling procedures ensured the study sample to be representative of the community-dwelling adults in the Central region of Singapore. This allows the findings to be generalised to this population. While the findings provide better understanding of cLBP in our population, several limitations should be acknowledged. Firstly, our study is restricted to cLBP whereas many other studies included both chronic and acute LBP. Hence, the actual prevalence of LBP in Singapore might be even higher; and the prevalence reported in our study and those reported in many other studies cannot be directly compared. Secondly, the cLBP status and settings of care seeking were based on self-reported data without using medical/professional assessment or hospital/clinic records as additional verification, hence, data used in this study might be subject to recall errors. Thirdly, although we did not observe significant differences in health outcomes between individuals who sought consultation/treatment and those did not, as the severity of cLBP was not collected, we could not infer whether the non-significant difference was due to the small sample size of participants with cLBP or the difference in severity of cLBP. Fourthly, as the study participants were only sampled from the community-dwelling adults in the Central region of Singapore, individuals who were hospitalised or institutionalised were not surveyed, therefore, the study findings could not be generalised to the entire adult population in Singapore. Hence, our findings should be interpreted with caution.

## Conclusions

The prevalence of chronic low back pain was 8.1% among the study population and 80.5% individuals with cLBP sought consultation/treatment in either primary care, SOC or TCM clinics. CLBP was associated with poorer physical function, more limitations and depressive symptoms, and lower health-related quality of life. The findings highlight the significant impact of cLBP on physical and mental health as well as health-related quality of life in a general adult population. Increased awareness on prevention, early and proper management of LBP, and rehabilitation policies are required to better tackle the burden of LBP at the population level.

## Supplementary Information

Supplementary Table S1.

## Data Availability

The datasets generated during and/or analysed during the current study are not publicly available due to the laws, rules and regulations stated in the “Advisory Guidelines for the Healthcare Sector”, but are available from the corresponding author on reasonable request.

## Abbreviations

cLBP: Chronic low back pain
CSSWs: Clerical, sales and service workers
DALYs: Disability-adjusted life years
EQ VAS: EQ visual analogue scale
GLM: Generalized linear models
GP: General practitioner
HRQoL: Health-related quality of life
LBP: Low back pain
Late-Life FDI: Late-life function and disability instrument
OR: Odds ratio
PA: Planning areas
PMETs: Professionals, managers, executives, and technicians
PHQ-9: 9-Item patient health questionnaire
PHI: Population health index
PTOCLs: Production and transport operators, cleaners & labourers
Q1: The first quartile (25th percentile)
Q3: The third quartile (75th percentile)
SD: Standard deviation
SOC: Specialist outpatient clinic
TCM: Traditional Chinese medicine
VAS: Visual analogue scale
YLDs: Years of life with disability
