# Supplementary material for: Chronic low back pain and its impact on physical function, mental health, and health-related quality of life: a cross-sectional study in Singapore
Source: Sci Rep. 2022 Nov 21;12:20040. doi: 10.1038/s41598-022-24703-7 (PMC9681885; doi:10.1038/s41598-022-24703-7)
Supplement: Supplementary file 1 — Supplementary Table S1. [file 41598_2022_24703_MOESM1_ESM.pdf]

Supplementary Table S1. Association between chronic low backpain and health outcomes using generalized linear models

Characteristics	Overall Function		p-value	Total Limitation		p-value	Depressive symptoms		p-value	EQ-5D Index		p-value	EQ VAS		p-value
	Coeff.	95% CI		Coeff.	95% CI		Coeff.	95% CI		Coeff.	95% CI		Coeff.	95% CI	
**Chronic low backpain**	-0.08	-0.10, -0.05	<.001	-0.04	-0.07, -0.01	.016	0.63	0.32, 0.94	<.001	-0.07	-0.09, -0.05	<.001	-0.10	-0.13, -0.07	<.001
**Female** (ref. male)	-0.04	-0.05, -0.02	<.001	0.001	-0.02, 0.02	.913	0.34	0.12, 0.55	.002	-0.02	-0.03, -0.01	.008	-0.002	-0.02, 0.02	.826
**Ethnicity** (ref. Chinese)															
Malay	-0.03	-0.06, -0.001	.043	-0.02	-0.06, 0.01	.227	0.26	-0.09, 0.60	.143	-0.01	-0.03, 0.02	.650	-0.01	-0.04, 0.02	.677
Indian	-0.02	-0.05, 0.003	.080	-0.03	-0.06, 0.002	.072	0.05	-0.23, 0.34	.720	0.001	-0.02, 0.02	.917	0.03	-0.002, 0.05	.067
Others	-0.06	-0.11, -0.01	.014	-0.08	-0.13, -0.02	.008	0.61	0.07, 1.15	.028	-0.05	-0.09, -0.02	.004	-0.02	-0.07, 0.03	.540
**Highest education level** (ref. no formal education)															
Primary	0.11	0.08, 0.14	<.001	0.08	0.04, 0.11	<.001	0.20	-0.16, 0.57	.279	0.02	-0.01, 0.04	.163	-0.001	-0.03, 0.03	.970
Secondary	0.14	0.11, 0.16	<.001	0.11	0.08, 0.14	<.001	0.15	-0.15, 0.45	.322	0.01	-0.01, 0.03	.295	0.004	-0.02, 0.03	.802
Post-secondary & above	0.20	0.17, 0.23	<.001	0.13	0.10, 0.17	<.001	0.25	-0.09, 0.59	.149	0.01	-0.01, 0.04	.243	-0.01	-0.04, 0.03	.689
**Marital status** (ref. single)															
Married	0.01	-0.01, 0.03	.214	0.01	-0.01, 0.04	.291	-0.40	-0.63, -0.17	.001	0.02	-0.001, 0.03	.058	0.02	0.001, 0.04	.040
Divorce/widowed	-0.07	-0.1, -0.04	<.001	-0.02	-0.05, 0.01	.247	0.03	-0.28, 0.34	.849	-0.03	-0.05, -0.01	.004	-0.004	-0.03, 0.02	.767
**Occupational groups** (ref. PMETs)															
CSSWs	0.01	-0.01, 0.04	.242	0.00	-0.03, 0.03	.966	-0.003	-0.28, 0.28	.983	0.01	-0.01, 0.03	.203	0.02	-0.01, 0.04	.160
PTOCLs	0.03	0.001, 0.06	.054	0.03	-0.01, 0.07	.096	0.06	-0.30, 0.41	.756	0.004	-0.02, 0.03	.714	0.01	-0.02, 0.04	.580
Others (unemployed &inactive)	-0.10	-0.13, -0.08	<.001	-0.04	-0.07, -0.02	.001	0.23	-0.04, 0.50	.089	-0.03	-0.04, -0.01	.003	-0.01	-0.03, 0.02	.487
**Live alone**	0.03	0.001, 0.05	.046	0.03	0.001, 0.06	.043	-0.44	-0.74, -0.13	.005	0.03	0.01, 0.05	.004	0.02	-0.01, 0.05	.156
**Self-reported money insufficiency**	-0.01	-0.04, 0.01	.247	-0.09	-0.12, -0.06	<.001	0.89	0.63, 1.16	<.001	-0.04	-0.05, -0.02	<.001	-0.08	-0.1, -0.05	<.001
**Smoking status** (ref. Never smoked)															
Former smoker	-0.02	-0.05, 0.004	.104	0.00	-0.04, 0.03	.772	0.04	-0.26, 0.34	.811	-0.02	-0.04, 0.003	.091	-0.005	-0.03, 0.02	.746
Current smoker	0.01	-0.01, 0.04	.272	0.01	-0.02, 0.04	.689	0.03	-0.27, 0.33	.843	-0.01	-0.03, 0.01	.233	-0.003	-0.03, 0.03	.849
**Alcohol misuse** (ref. No)	0.03	0.01, 0.05	.001	0.03	0.003, 0.05	.025	0.44	0.22, 0.65	<.001	0.01	-0.01, 0.02	.366	-0.01	-0.03, 0.01	.226
**Number of chronic morbidities** (ref. 0)															
1	-0.03	-0.05, -0.01	.007	0.002	-0.02, 0.03	.856	0.44	0.20, 0.68	<.001	-0.02	-0.03, -0.001	.040	-0.03	-0.05, -0.004	.022
2 and over	-0.14	-0.16, -0.12	<.001	-0.05	-0.07, -0.02	<.001	0.79	0.56, 1.02	<.001	-0.06	-0.07, -0.04	<.001	-0.06	-0.08, -0.03	<.001

Coeff.: beta coefficient, 95% CI: 95% confidence interval. Age was excluded from the model due to collinearity.
